# The major secreted protein Msp1/p75 is *O*-glycosylated in *Lactobacillus rhamnosus *GG

**DOI:** 10.1186/1475-2859-11-15

**Published:** 2012-02-01

**Authors:** Sarah Lebeer, Ingmar JJ Claes, Crina IA Balog, Geert Schoofs, Tine LA Verhoeven, Kris Nys, Ingemar von Ossowski, Willem M de Vos, Hanne LP Tytgat, Patrizia Agostinis, Airi Palva, Els JM Van Damme, André M Deelder, Sigrid CJ De Keersmaecker, Manfred Wuhrer, Jos Vanderleyden

**Affiliations:** 1 Centre of Microbial and Plant Genetics, K.U.Leuven, Kasteelpark Arenberg 20, box 2460, B-3001 Leuven, Belgium; 2 Department of Bioscience Engineering, University of Antwerp, Groenenborgerlaan 171, B-2020 Antwerp, Belgium; 3 Biomolecular Mass Spectrometry Unit, Department of Parasitology, Leiden University Medical Center, Leiden, The Netherlands; 4 Cell Death Research & Therapy laboratory, Department Molecular and Cell Biology, Faculty of Medicine, K.U.Leuven, Herestraat 49, box 901, B-3000, Belgium; 5 Department of Veterinary Biosciences, University of Helsinki, P.O. Box 66, FIN-00014, Helsinki, Finland; 6 Department of Molecular Biotechnology, Ghent University, Ghent, Belgium

**Keywords:** Probiotic, glycoprotein, bacterial *O-*glycosylation, Akt signaling, peptidoglycan hydrolase

## Abstract

**Background:**

Although the occurrence, biosynthesis and possible functions of glycoproteins are increasingly documented for pathogens, glycoproteins are not yet widely described in probiotic bacteria. Nevertheless, knowledge of protein glycosylation holds important potential for better understanding specific glycan-mediated interactions of probiotics and for glycoengineering in food-grade microbes.

**Results:**

Here, we provide evidence that the major secreted protein Msp1/p75 of the probiotic *Lactobacillus rhamnosus *GG is glycosylated. Msp1 was shown to stain positive with periodic-acid Schiff staining, to be susceptible to chemical deglycosylation, and to bind with the mannose-specific Concanavalin A (ConA) lectin. Recombinant expression in *Escherichia coli *resulted in a significant reduction in molecular mass, loss of ConA reactivity and increased sensitivity towards pronase E and proteinase K. Mass spectrometry showed that Msp1 is *O-*glycosylated and identified a glycopeptide TVETPSSA (amino acids 101-108) bearing hexoses presumably linked to the serine residues. Interestingly, these serine residues are not present in the homologous protein of several *Lactobacillus casei *strains tested, which also did not bind to ConA. The role of the glycan substitutions in known functions of Msp1 was also investigated. Glycosylation did not seem to impact significantly on the peptidoglycan hydrolase activity of Msp1. In addition, the glycan chain appeared not to be required for the activation of Akt signaling in intestinal epithelial cells by Msp1. On the other hand, examination of different cell extracts showed that Msp1 is a glycosylated protein in the supernatant, but not in the cell wall and cytosol fraction, suggesting a link between glycosylation and secretion of this protein.

**Conclusions:**

In this study we have provided the first evidence of protein *O-*glycosylation in the probiotic *L rhamnosus *GG. The major secreted protein Msp1 is glycosylated with ConA reactive sugars at the serine residues at 106 and 107. Glycosylation is not required for the peptidoglycan hydrolase activity of Msp1 nor for Akt activation capacity in epithelial cells, but appears to be important for its stability and protection against proteases.

## Background

The bacterial cell surface mediates many interactions between bacteria and their changing and sometimes harsh environment [1,2]. Diverse selective pressures are acting on bacterial cell surface molecules, resulting in various adaptations of their chemical and structural composition. This is especially true for the wide array of glycans that often can decorate bacterial cell walls and which are collectively called the bacterial glycome [3]. Cell wall components encompassing the bacterial glycome can include lipopolysaccharides in Gram-negative bacteria, glycosylated teichoic acids in Gram-positive bacteria and peptidoglycan, exopolysaccharides, capsular polysaccharides, glycolipids as well as glycoproteins in both types of bacteria. Bacterial protein glycosylation has long been overlooked, however *O- *and *N-*linked protein glycosylation systems are increasingly being documented among pathogenic bacteria [4-7].

Overall interest in studying bacterial glycoproteins has grown steadily during the past decade, with most reports focused on the various surface structures (e.g., flagellae, pili) related to pathogenesis [5]. In contrast, the glycoproteome of beneficial microbes (commensals and probiotics) has been much less documented so far. Nevertheless, knowledge about protein glycosylation in beneficial microbes holds important potential for the development of 'safe' glycoengineering purposes, such as enhancing the stability and pharmacokinetic properties of therapeutic proteins [8,9] and the design of specific immunomodulatory agents since glycans can mediate very specific interactions, especially in microbe-host signaling [10,11]. Probiotic bacteria, such as various lactobacilli and bifidobacteria with documented health-promoting capacities, are among the best candidates for these purposes. Glycosylation of proteins was previously suggested in *Lactobacillus acidophilus *[12] and *Lactobacillus plantarum *[2], but without detailed analyses. Owing to its frequent use in clinical trials [13], we study *Lactobacillus rhamnosus *GG (LGG) and use it as a model probiotic bacterium for genetic and biochemical investigations on the functional importance of the cell surface properties of such beneficial strains. Single molecule force spectroscopy (SMFS) experiments with lectin-functionalized atomic force microscopy tips have suggested the presence of two major types of surface glycans in LGG [14]. The longest and most abundant polysaccharides are galactose-rich, and correspond with the galactose-rich exopolysaccharide (EPS) molecules [15]. The shorter Concanavalin A (ConA)-reactive glycans are yet unknown [14]. In this current study, we identified the Msp1 (or p75) protein of LGG as a ConA-reactive glycoprotein, investigated its glycosylation site(s) and analyzed the functionality of the glycan component of this protein for some of its documented biological activities. This protein, identified previously as a major secreted protein of LGG, has been shown to have anti-apoptotic and growth-promoting effects in intestinal epithelial cells [16]. Recently, we showed that this protein shows peptidoglycan hydrolase activity with D-glutamyl-L-lysyl endopeptidase specificity and is important for daughter cell separation of LGG [17]. In our present study of Msp1, we provide - to the best of our knowledge - the first example of an *O-*glycosylated protein in the probiotic LGG.

## Results

### Aberrant electrophoretic migration pattern of Msp1

**Figure 1:**
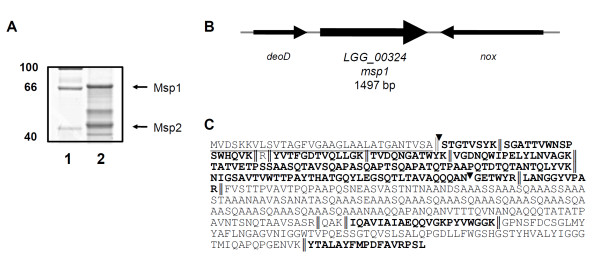
**Aberrant migration of Msp1 on SDS-PAGE compared to theoretical predictions**. **(A) **SDS-PAGE analysis of LGG's secreted proteins present in spent culture supernatant after overnight growth in AOAC medium. The proteins in lane 1 correspond to the molecular mass standard (Candy Cane). Lane 2 corresponds to the secreted proteins of LGG WT. Arrows indicate the major secreted proteins of LGG. Msp1 was confirmed by Edman degradation and MS/MS to correspond to LGG_00324 (YP_003170070). Msp2 was confirmed by Edman degradation and MS/MS to correspond to LGG_00031 (YP_003169777). The gels are stained with Sypro^® ^Ruby. **(B) Genomic context of the *msp1 *gene (*LGG_00324)*. (C) Amino acid sequence of Msp1**. The underlined amino acids indicate the signal peptide that is cleaved off after secretion. Additionally, the theoretical trypsin digest is depicted (indicated by ||). The theoretical mass and pI after cleavage of the signal peptide are respectively 6.06 and 46779 Da. Peptides that were experimentally identified by Edman degradation or MS/MS analysis are indicated in bold. As trypsin digestion yields long peptide fragments, this complicated the identification of multiple peptides. The truncated form of Msp1 which is expressed in the *msp1 *mutant CMPG10200 is indicated in between the triangles (▼).

**Figure 2:**
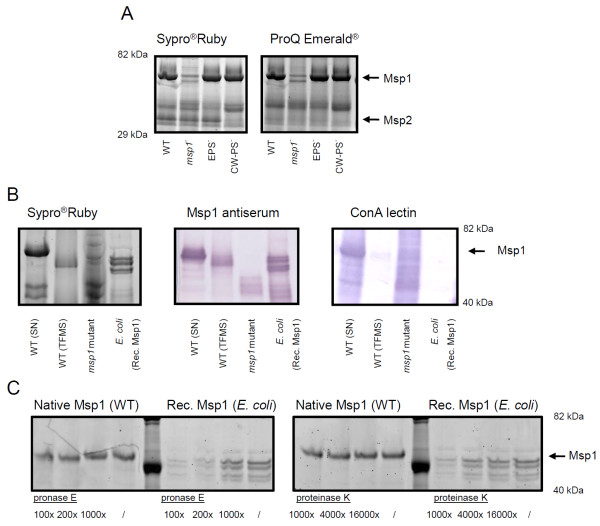
**Identification of Msp1 as a glycoprotein**. **(A) **SDS-PAGE analysis of LGG's secreted proteins present in spent culture supernatant after overnight growth in AOAC medium. The secreted proteins of LGG WT, *msp1 *mutant, EPS mutant CMPG5351 and CW-PS mutant CMPG5413 are included. Arrows indicate the major secreted proteins of LGG Msp1 and Msp2 confirmed by Edman degradation and MS/MS. The gels are stained with Sypro^® ^Ruby and ProQ Emerald glycoprotein stain. **(B) **Comparative analysis of the glycosylation of Msp1 in spent culture supernatant of LGG wild-type before and after treatment with TFMS, and after recombinant expression in *E. coli*. The *msp1 *mutant CMPG10200 was included as a control. This mutant expresses a C-terminally truncated form of Msp1 and lacks the enzymatic active NlpC/P60 domain [17]. SDS-PAGE gels were compared by ConA lectin blotting and blotting with Msp1 specific antiserum. (C) Comparison of the sensitivity of recombinant non-glycosylated Msp1 purified from *E. coli *(rec. Msp1) and native glycosylated Msp1 purified from LGG WT (glyc. Msp1) towards pronase E and proteinase K. Protein samples were incubated for 1 h at 37°C with the indicated diluted preparations of proteases (Sigma). SDS-PAGE gels were stained with Sypro^® ^Ruby (Invitrogen). Images were scanned with Typhoon 9400 (GE Healthcare).

Msp1, described previously as the p75 protein [16], is present in abundant amounts in the spent culture supernatant of LGG. Interestingly, this protein appears to have an aberrant migration pattern when analyzed by SDS-PAGE and is detected with a significantly higher molecular mass (70-75 kDa) (Figure 1A) than the expected mass (46.8 kDa) for a protein with a N-terminal secretion signal (Figure 1B-C). Previously, this protein was named (i.e. p75) based on its size on SDS gels. Because of the apparent inconsistency in molecular mass, we have designated this protein according to its relative abundance as Msp1 (as in the Major secreted protein). While our electrophoretic results are strongly suggestive of post-translational modification, part of the observed discrepancy in molecular mass might also be due to the disproportionately high number of alanine residues (~23% content) in the primary structure of this protein (Figure 1C). Alanine has a low SDS-binding capacity and is known for a high helical propensity causing slow gel migration for proteins with high alanine content [18]. However, we found that a *msp1 *mutant CMPG10200 [17] (Table 1), expressing a truncated form of Msp1 that lacks a 311-residue C-terminal segment encompassing a large proportion of alanines, still migrates with a larger (~45 kDa) (Figure 2B) than expected size (16.4 kDa) (Figure 1C). Contrastingly, the other major secreted protein of LGG (encoded by *LGG_00031 *), here renamed as Msp2 but also known previously as p40 [16], has a predicted molecular mass of 39.7 kDa that corresponds well with its observed migration pattern on SDS gels, suggesting that this protein has not undergone major post-translational modifications (Figure 1A).

**Table 1:** *Lactobacillus *strains used in this study

*Lactobacillus*	Strain number	*Relevant characteristics*	*Reference*
*L. rhamnosus *GG (LGG)	ATCC53103	Human isolate; wild-type (WT)	ATCC
	CMPG10200	Msp1/p75 knock-out mutant, Ery^R^	[17]
	CMPG5413	Deficient in major cell wall polysaccharides (CW-PS); Tc^R^	[29-31]
	CMPG5351	*welE *knock-out mutant; deficient in long-galactose-rich EPS; Tc^R^	[15]
*L. rhamnosus*	ATCC7469	Dairy isolate; type strain	BCCM/ATCC
*L. rhamnosus*	GR-1	Human vaginal isolate	[32]; Chr. Hansen
*L. casei*	ATCC334	Dairy isolate, type strain	BCCM/ATCC
*L. casei*	ATCC393	Dairy isolate	BCCM/ATCC
*L. casei*	DN-114001	Dairy isolate	Danone (Actimel)

### Msp1 visually stains as a glycosylated protein

As the first means to identify any potential carbohydrate modifications on Msp1, proteins in the supernatant of spent cultures of LGG were separated by SDS-PAGE and then treated with a stain that specifically reacts with periodate-oxidized carbohydrate groups (Pro-Q^® ^Emerald 488 glycoprotein stain). As shown in Figure 2A, a ~70-kDa stained band was detected and which we consider to be glycosylated Msp1. However, given that this glycoprotein stain might also cross-react with cell wall polysaccharides, a galactose-rich EPS deficient mutant CMPG5351 [15] and another type of cell wall polysaccharide (CW-PS)-deficient mutant CMPG5413 [14] of LGG were included as controls. As indicated by the staining results (Figure 2A), CMPG5351 and CMPG5413 spent culture supernatants both also displayed a similar-sized band (~70 kDa) as wild-type LGG. Msp2 exhibited no staining above the background level, thus further suggesting that this protein is unlikely to be glycosylated (Figure 2A). To confirm the covalent nature of the carbohydrate modification, and so to exclude any possibility that glycan moieties are merely associated with the protein, we treated Msp1 with the chemical deglycosylating agent trifluoromethanesulfonic acid (TFMS) and were able to observe a clear shift in band migration relative to untreated protein on SDS-PAGE (Figure 2B). To provide additional evidence for glycosylated Msp1, we expressed the *msp1 *gene in a non-glycosylating *E. coli *host (BL21(DE3)/pLysS) and found that this recombinant form is significantly reduced in size when compared to native Msp1 on SDS-PAGE (Figure 2B). Interestingly, several discrete and smaller-sized bands were also associated with the recombinant Msp1 sample and subsequently confirmed to represent truncated Msp1 protein after each band was analyzed by Edman degradation, mass spectrometry (MS) (data not shown), and Western blotting with anti-Msp1 serum (Figure 2B). The presence of these truncated forms suggests that the recombinant-produced Msp1 is less stable, which possibly might be related to the alternate codon usage in *E. coli*. However, given that the non-glycosylated recombinant form appeared to be more sensitive to pronase E and proteainase K than native Msp1 (Figure 2C), this is more likely attributed to the Msp1 glycosylated protein being more protease-resistant.

### Identification of Msp1 as a ConA-reactive glycoprotein

To further confirm the glycoprotein nature of Msp1, lectin blotting experiments were carried out. Interestingly, the mannose- and glucose-specific lectin ConA [19] reacted positively with Msp1 (Figure 2B, third panel). Following TFMS treatment or recombinant expression in *E. coli *BL21(DE3)/pLysS, this reactivity was no longer detected for Msp1. These data were further confirmed by testing other lectins that have overlapping specificities, i.e. GNA from *Galanthus nivalis *and HHA from *Hippeastrum hybrid *(data not shown). Given that all the above lectins interact with terminal mannose residues [20], other lectins with different specificities, such as fucose (from *Ulex europaeus*) and sialic acid (MAA from *Maackia amurensis *and SNA-I from *Sambucus nigra*), were also used but these failed to react (data not shown). Monosaccharide component analysis confirmed the presence of mannose in this glycoprotein and absence of fucose and sialic acid (data not shown). The C-terminally truncated form of Msp1 of the CMPG10200 mutant appeared to be still reactive with ConA (Figure 2B, third panel), suggesting that the ConA-reactive glycosylation site(s) are confined to the N-terminal part.

### Msp1 is *O-*glycosylated at S_106_-S_107_

**Figure 3:**
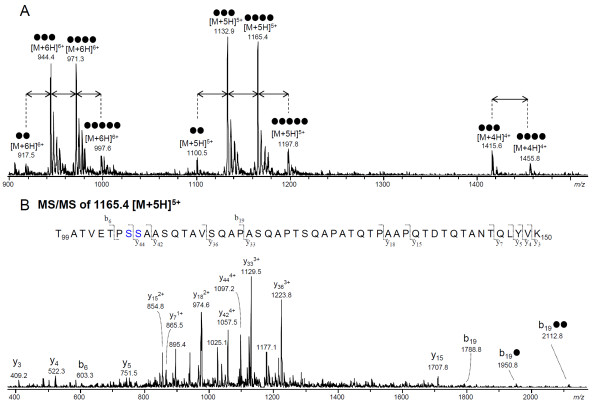
**Analysis of tryptic Msp1 glycopeptides by mass spectrometry**. (A) Sum mass spectrum of the elution range of tryptic glycopeptides T_99_K_150 _carrying two to five hexose residues. Glycopeptides were detected with four, five, and six charges. (B) Ion trap-MS/MS of T_99_K_150 _carrying four hexoses ([M+5H]^5+ ^at *m/z *1165.4). The MS/MS spectrum was acquired by nano-LC-MS/MS of the total tryptic digest of Msp 1. The Ser-Ser motif which was found to be substituted by hexoses is highlighted in blue. Filled circle indicates hexose.

Glycosylation of Msp1 was further analyzed by MS of (glyco-)peptides. Gel bands containing Msp1 were subjected to reduction, alkylation, and trypsin treatments and the resulting peptides were analyzed by reversed phase-LC-ion trap-MS/MS. Evaluation of fragmentation spectra together with searches of public databases (NCBI) gave partial coverage of the Msp1 protein (gi199599310). Most peptides with sizes larger than 600 Da and smaller than 8000 Da were identified by either database searching or manual interpretation (Table 2). The manual interpretation of the LC-MS/MS data revealed glycopeptide clustering that appears as [M+4H]^4+^, [M+5H]^5+ ^, and [M+6H]^6+ ^ions (Figure 3A). As was determined by tandem MS, glycopeptides differed in the number of hexoses attached to the peptide moiety, which was reflected by a mass difference of 162 Da. As an example, the fragmentation spectrum of *m/z *1165.4 [M+5H]^5+ ^is shown in Figure 3B. The peptide species is identified as T_99_ATVETPSSAASQTAVSQAPASQAPTSQAPATQTPAAPQTDTQTANTQLYVK_150 _and was found to carry four hexoses. The y ions as well as the b_6 _ion confirm the identity of these glycopeptides. While most fragment ions including all y ions were observed without the hexoses being attached, the b_19 _cleavage product was registered without hexoses (*m/z *1788.8), with 1 hexose (*m/z *1950.8), and with 2 hexoses (*m/z *2112.8), indicating that at least part of the hexoses is linked to the N-terminal portion of this peptide fragment. These data demonstrate that the glycosylation on Msp1 varies from two to five hexoses linked to T_99_K_150_.

**Table 2:** Sequence coverage of Msp1.Tryptic peptides were registered by RP-LC-ion trap-MS/MS. Filled circles indicate hexoses

(Glyco)peptide	Registered signal (*m/z*)	Calculated mass/determined mass	Mascot score
S_33_K_40_	Not detected	841.42/-	
S_41_K_56_	595.48 [M+3H]^3+^	1783.41/1783.44	60
R_57_	Not detected	174.11/-	
Y_58_K_81_	720.90 [M+2H]^2+^	1439.78/1439.80	90
T_71_K_81_	641.78 [M+2H]^2+^	1281.54/1281.56	68
V_82_K_98_	639.34 [M+3H]^3+^	1914.99/1915.02	46
T_99_K_150_	Not detected	5170.51/-	
T_99_K_150 _●●	1374.56 [M+4H]^4+^ 1099.81 [M+5H]^5+^ 916.81 [M+6H]^6+^	5494.62/5494.35	Manually assigned
T_99_K_150 _●●●	1415.06 [M+4H]^4+^ 1132.31 [M+5H]^5+^ 943.75 [M+6H]^6+^	5656.67/5656.43	Manually assigned
T_99_K_150 _●●●●	1455.55 [M+4H]^4+^ 1164.69 [M+5H]^5+^ 970.75 [M+6H]^6^	5818.72/5818.38	Manually assigned
T_99_K_150 _●●●●●	1496.25 [M+4H]^4+^ 1197.13 [M+5H]^5+^ 997.88 [M+6H]^6+^	5980.77/5980.98	Manually assigned
N_151_R_193_	1168.50 [M+4H]^4+^	4669.25/4669.0	Manually assigned
L_194_R_203_	509.24 [M+2H]^2+^	1016.46/1016.48	49
F_204_R_378_	Not detected	15931.57/-	
Q_379_K_381_	Not detected	345.20/-	
I_382_K_402_	752.78 [M+3H]^3+^	2255.32/2255.34	92
G_403_K_481_	Not detected	8257.93/-	
Y_482_L_498_	989.46 [M+2H]^2+^	1976.90/1976.92	90

**Figure 4:**
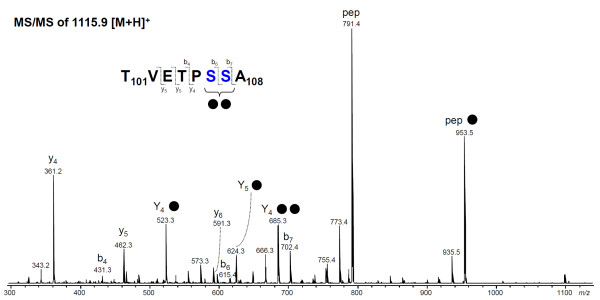
**Tandem mass spectrometry of the proteinase K-generated peptide T_101_VETPSSA_108 _carrying two hexoses**. The MS/MS spectrum of *m/z *1115.9 [M+H]^+ ^was acquired by graphitized carbon nano. The Ser-Ser motif which was found to be substituted by hexoses is highlighted in blue. Filled circle indicates hexose.

Since the very large tryptic peptides of Msp 1 (F_204_R_378_, MW15931.57 and G_403_K_481_, MW 8257.93) could not be detected by MS, they were first digested by proteinase K and then further subjected to analysis for glycosylation. However, because proteinase K treatment is known to often result in glycopeptides made up of small peptide moieties (up to 10 amino acid residues) [21] that show no or insufficient retention in reverse phase LC, further analysis was instead performed by graphitized carbon-LC-ion trap-MS/MS. As a result, a group of glycopeptides could be registered that were substituted with one or two hexose residues. As an example, the fragmentation of peptide T_101_VETPSSA_108 _substituted with 2 hexose residues is shown in Figure 4. A glycosylated y_4 _fragment ion containing two hexose residues (*m/z *685.4) allows the assignment of the hexoses to the S_106_-S_107 _dipeptide. The other glycopeptides T_104_PSSA_108_, T_101_VETPSSA_108 _and T_101_VETPSSAA_109 _or A_100_TVETPSSA_108 _with one or two hexose residues also covered the same S_106_-S_107 _dipeptide. No glycopeptides were registered covering parts of the large tryptic peptides of F_204_R_378 _and G_403_K_481_.

### ConA reactivity correlates with presence of the S_106_-S_107 _dipeptide in *L. rhamnosus *but not in *L. casei *strains

**Figure 5:**
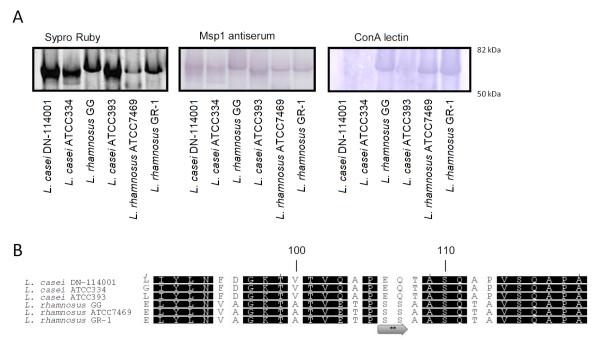
**Comparative analysis of the glycosylation of Msp1 in *L. rhamnosus *and *L. casei *strains**. **(A) **Supernatant proteins from different *L. rhamnosus *and *L. casei *strains were run on SDS-PAGE and stained with Sypro^® ^Ruby, and transferred to PVDF membranes for Western blot analysis with Msp1 antiserum or ConA lectin. **(B) **Multiple alignment of part of the Msp-1 like proteins in different *L. casei *and *L. rhamnosus *strains. The corresponding *msp1 *genes were obtained by sequencing after PCR as described in the Methods section. The glycosylation site on the S_106_-S_107 _dipeptide determined by MS/MS for LGG is indicated with asterisks.

Multiple-sequence alignment analysis of the primary structure of Msp1 (p75) from different *L. rhamnsosus *and *L. casei *strains suggested that the amino acid residues of the T_104_PSSA_108 _fragment covering the S_106_-S_107 _dipeptide in Msp1 of *L. rhamnosus *strains are replaced with A_104_PEQT_108 _in several *L. casei *strains [22]. To determine whether this is a factor in ConA reactivity, parallel experiments were performed involving ConA and anti-Msp1 serum blotting (Figure 5A) with spent culture supernatant proteins from different *L. casei *and *L. rhamnosus *strains that were obtained from our laboratory collection (Table 1). All samples tested from *L. rhamnosus *and *L. casei *appeared to exhibit a protein that is recognized by the antiserum raised against recombinant LGG Msp1, although the molecular mass of the Msp1-like proteins in the *L. casei *strains tested seems to be smaller than Msp1 in the *L. rhamnosus *strains (Figure 5A). Results from blotting experiments with ConA indicated that only the Msp1 proteins of the *L. rhamnosus *strains confirmed to have the S_106_-S_107 _dipeptide (Figure 5B) display the capacity to interact with ConA (Figure 5A).

### Msp1 glycosylation is not crucial for Akt activation of LGG Msp1 in Caco-2 cells

**Figure 6:**
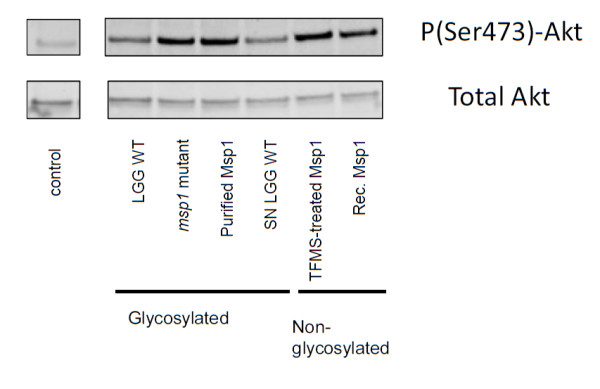
**Impact of Msp1 glycosylation on Akt activation in intestinal epithelial cells**. Caco-2 cells were co-incubated with *L. rhamnosus *GG wild-type (10^7 ^CFU/ml), *msp1 *mutant CMPG10200 (10^7 ^CFU/ml), glycosylated Msp1 after purification with affinity chromatography and ConA sepharose (ca. 100 ng/ml), Msp1 partially purified from spent culture supernatant (SN) (ca. 10 ng/ml), chemically deglycosylated Msp1 after TFMS treatment (ca. 100 ng/ml) and recombinantly (rec.) expressed Msp1 after purification from *E. coli *(ca. 100 ng/ml). Subsequent Akt activation was monitored by Western blot analysis of cellular lysates with anti-P-Akt and ant-Akt antibodies. Data are representative for 2 separate experiments.

Previously, Msp1 (or p75) was identified by Polk and co-workers as a soluble protein of LGG that can regulate intestinal epithelial cell survival and growth via Akt signaling [16]. Here, we investigated what role the glycan chain of Msp1 plays in Akt activation in the Caco-2 intestinal epithelial cell line. To do this, both purified and TFMS-deglycosylated Msp1 and recombinantly produced Msp1 were used. As shown in Figure 6, no significant difference in Akt activation was found for the glycosylated and non-glycosylated forms. The *msp1 *mutant still showed Akt activation, which likely reflects the presence of Msp2, which is a stronger activator of Akt signaling than Msp1 [16].

### Msp1 glycosylation is not crucial for peptidoglycan hydrolase activity of Msp1

**Figure 7:**
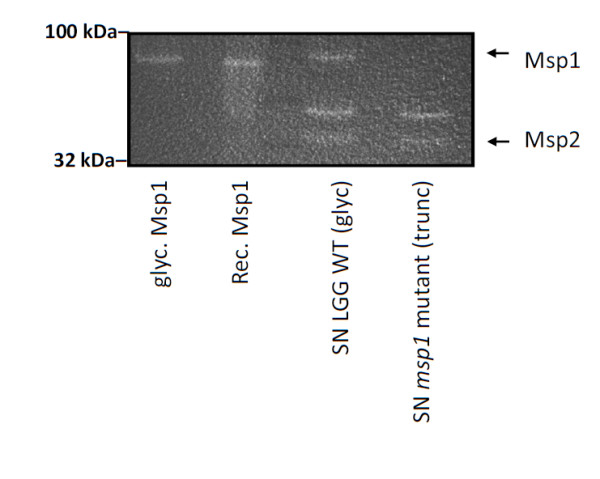
**Impact of Msp1 glycosylation on peptidoglycan hydrolase activity**. The cell wall hydrolyzing capacity of glycosylated (glyc.) and recombinant (rec.) non-glycosylated Msp1 was determined with *msp1 *mutant CMPG10200 cells as substrate. Supernatant (SN) samples from LGG wild-type (WT) and the *msp1 *mutant CMPG10200 were also loaded on the zymogram gels. The gels were incubated overnight at 37°C in phosphate buffer containing 1 mM DTT supplemented with the different Msp1 samples and visualized by methylene blue staining as described in materials and methods.

Recently, we demonstrated that Msp1 is a peptidoglycan hydrolase with D-glutamyl-L-lysyl endopeptidase activity [17]. Here, we aimed to determine whether glycosylation is required for the peptidoglycan hydrolase activity by comparing the native (glycosylated) and recombinant (non-glycosylated) forms of Msp1. Our zymogram analyses indicated that Msp1 in spent culture supernatant and both purified native and recombinant Msp1 all share the same level of activity (Figure 7). However, the *msp1 *mutant CMPG10200, which expresses a truncated Msp1 protein that lacks the important catalytic NlpC/p60 domain, was not active in agreement with our previous results [17].

### Msp1 glycosylation is linked with localization in the supernatant

**Figure 8:**
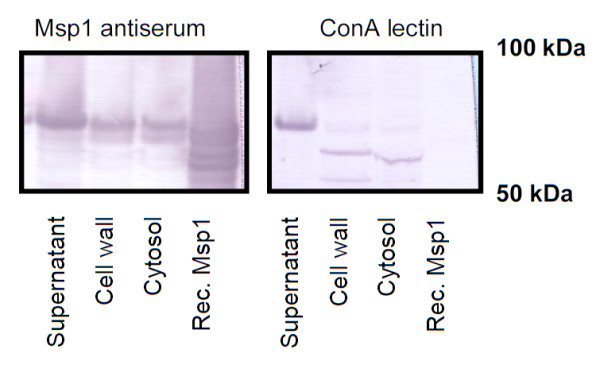
**Relationship between Msp1 glycosylation and cellular location**. LGG wild-type was grown for 24 h in AOAC medium and proteins were isolated from spent culture supernatant, the cell wall and cytosol fraction as described in Materials and Methods. Samples were run on SDS-PAGE and blot with Msp1 antiserum or ConA lectin. For comparison, non-glycosylated recombinant Msp1 (rec. Msp1) was included.

To ascertain whether a link exists between the cellular location and the glycosylation of Msp1, we isolated proteins from the spent culture supernatant, cell wall, and cytosol, and performed parallel blotting experiments with ConA and anti-Msp1 serum. As our results indicate, although Western blotting with Msp1-specific antiserum confirmed the presence of Msp1 in all cellular fractions, the cytosolic and cell wall fractions showed no significant reactivity with ConA, unlike the spent culture supernatant fraction which did exhibit strong ConA reactivity (Figure 8).

## Discussion

In this study, we have reported the presence of an *O-*glycosylated protein in a probiotic *Lactobacillus *strain. LGG's major secreted protein Msp1, which we renamed from p75 [16], has been shown previously to mediate intestinal cell homeostasis through Akt signaling pathways, but also exhibits an interesting aberrant gel migration pattern. To provide evidence for the glycosylation of Msp1, we used a combination of several standard glycosylation detection methods, which include periodate-oxidation, chemical deglycosylation with TFMS, and lectin blotting with ConA, which is specific for mannose or glucose residues. Moreover, we demonstrated that a recombinant form of Msp1, which was produced in a non-glycosylating *E. coli *host, has a significantly reduced molecular mass and cannot bind to ConA. In addition, we also found that recombinant Msp1 was less stable than the native form. Mass spectrometry experiments confirmed that Msp1 is indeed *O-*glycosylated and identified a glycopeptide fragment (T_101_VETPSSA_108_) that displays at least two hexose moieties on S_106_-S_107_. These data do not allow distinguishing between the following possibilities: the occurrence of two single hexoses attached to serine residues, or the presence of a disaccharide substituent. In addition, larger glycopeptides species where observed that covered the same S_106_-S_107 _glycosylation site(s) and carried up to 5 hexoses. The obtained fragmentation spectra did not allow the assignment of the hexoses to specific sites. The observed glycopeptides T_99_-K_150 _are rich in serine and threonine residues, which both represent potential hexose attachment sites. Although some y ions representing only the peptide backbone have been detected in the MS/MS spectra of these glycopeptides, the existence of di-, tri-, tetra- or pentahexosyl units attached to Msp 1 remains speculative since no corresponding glycan oxonium ions were observed (e.g. no [hexose_2_+H]^+ ^signal at *m/z *325). However, based on the fragmentation spectrum of the tryptic glycopeptides, the occurrence of such oligosaccharide moieties cannot be excluded. With regard to the nature of the hexose units, the above-mentioned Con A binding in combination with the binding to GNA from *Galanthus nivalis *and HHA from *Hippeastrum hybrid *(mannose specific lectins) indicate these to be mannose residues. Additional research is required to completely characterize the O-glycans and exact glycosylation sites of Msp1.

Interestingly, in the homologous forms of Msp1 in several *L. casei *strains tested, which do not react with ConA, these serine residues are missing, thus underscoring their importance as glycosylation sites in Msp1 of LGG (and other *L. rhamnosus *strains) and the basis for ConA reactivity. However, this does not exclude the possibility that the Msp1-like proteins of *L. casei *are glycosylated at other sites with other sugars. In fact, preliminary lectin blotting experiments suggest glycosylation of Msp1 in *L. casei *with galactose or N-acetylgalactosamine residues. Thus, our results are strongly suggestive of a species-specific glycosylation mechanism for Msp1 in different *L. rhamnosus *bacteria.

Subsequently, we investigated the role of the glycan chain in functions established previously for Msp1 of *L. rhamnosus *GG, which include the activation of Akt signaling in intestinal epithelial cells [16] and a peptiglycan hydrolase activity [17]. Our results indicate that glycosylation of Msp1 does not appear to be crucial for either of these two functions. The lack of a direct role for the glycosylation of Msp1 in its activation of Akt signaling is not surprising, given that Msp2, as an apparently non-glycosylated protein (this study), can also activate Akt in intestinal epithelial cells and at lower concentrations than does Msp1 (p75) [16]. However, this does not exclude a possible modulating role for the Msp1 glycan chains in Akt activation. For instance, these glycan components might still act to shield host cell receptors and, in so doing, possibly cause a reduction in the capacity of Msp1 and IECs to interact, thus modulating the extent of Akt signaling. The finding that the specific ConA-reactive glycosylation of Msp1 is not required for peptidoglycan hydrolase activity is in full agreement with the fact that the *L. rhamnosus *GG and *L. casei *BL23 Msp1-like proteins both exhibit identical enzymatic properties [17,23], while the glycosylation site T_101_VETPSSA_108 _in *L. rhamnosus *GG Msp1 is replaced with T_101_VQAPEQY_108 _in the Msp1-like protein in *L. casei *BL23 [22] and the Msp1 proteins of the *L. casei *strains used in our study do not show ConA reactivity.

The protein glycosylation machinery of *L. rhamnosus *GG is currently unknown. Our data related to the cellular localization of Msp1 in *L. rhamnosus *GG reveal a possible link between the glycosylation of Msp1 and its release into the surrounding milieu. Furthermore, lectin blotting experiments indicate that other secreted proteins, including the putative cell wall hydrolase encoded by *LGG_02225 *might be glycosylated, suggesting a more global mechanism (data not shown). This would be in agreement with the fact that there appear to be no neighboring genes of the *msp1 *gene that putatively encode for glycosyltransferases, in contrast to dedicated protein glycosylation systems [7]. Our future experiments will include determining the exact glycosylation mechanisms and the role of glycosylation of Msp1 and related proteins in the control of their activity and localization. The function of glycosylation for Msp1 might possibly be similar to that which was reported for the HMW1 protein in *Haemophilus influenza*, which includes the protection against premature degradation and the tethering of protein to the cell surface [24]. In addition, various specific C-type lectin receptors of the innate immune system, such as the dendritic cell-specific intercellular adhesion molecule-3-grabbing non-integrin DC-SIGN [10,25], show a sugar specificity that overlaps with ConA. Future experiments are also aimed at elucidating the role of Msp1 glycosylation in innate immune signaling.

## Conclusions

In this study we have provided convincing evidence that Msp1 (p75) of the probiotic *L. rhamnosus *GG is *O-*glycosylated with ConA-reactive sugars at the serine residues in T_101_VETPSSA_108 _once released into the external surroundings. Further studies are now required to determine the exact glycan chain, mechanism and functional importance of glycosylation in Msp1. Nonetheless, the first identification of a glycosylation site in a probiotic-related protein provides interesting opportunities in a safe and food grade bacterium for glyco-engineering purposes such as targeting specific immune cells or enhancing the stability.

## Methods

### Bacterial strains and culture conditions

Lactobacilli (Table 1) were grown at 37°C in MRS medium (Difco) or in Lactobacilli AOAC medium (Difco) in non-shaking conditions. CMPG10200 was grown in the presence of 5 μg/ml erythromycin for stable maintenance of the integrative plasmid. In addition, *Escherichia coli *strains TOP10 [*F- mcrA Δ(mrr-hsdRMS-mcrBC) F80lacZ ΔM15 ΔlacX74 recA1 araD139 Δ(ara-leu)7697 galU galK rpsL (StrR) endA1 nupG*] and BL21(DE3)/pLysS [*F- ompT hsdSB(rB- mB-) gal dcm (DE3) pLysS (CamR)*] were used for plasmid preparation and protein expression, respectively. *E. coli *was routinely grown in Luria-Bertani (LB) medium at 37°C with agitation and was supplemented with 50 μg/ml kanamycin when required.

### Molecular cloning

Standard DNA protocols were used for molecular cloning and related procedures as described previously [18]. Sequencing of the *msp1 *gene from different *L. rhamnosus *and *L. casei *strains (Table 1) was performed on PCR products obtained by colony PCR with primers Pro-0997 (5'ATGAATTCACAGGGACGGTCAGTTACAAATCC-3', EcoRI site underlined) and Pro-0998 (5'-ATGAATTCGTTGTTCGGCAATGGCAATCACTG-3', EcoRI site underlined) for *L. rhamnosus *and Pro-0679 (5'-AGAAAGTATTGTCAGTAACGGCAGG-3') and Pro-0680 (5'-TTGGCAAGCCGATACCATGTTTCAC-3') for *L. casei *strains by Macrolon (The Netherlands). These DNA sequences were translated to amino acid sequence and a multiple alignment around the S_106_-S_107 _dipeptide of LGG was constructed using the Geneious Pro™ software.

### Production of purified recombinant Msp1 protein

The coding sequence for *msp1 *(*LGG_00324*) not including the sequence for the N-terminal signal peptide was amplified by PCR from LGG genomic DNA by means of two flanking 5'- and 3'-end oligoprimers, one with an EcoRI restriction site (5'- TACCGTTTCGAATTCGACAGGGACGGTC-3', EcoRI site is underlined]) and another with an XhoI restriction site (5'- GATCATTAAGCCTCGAGTGACGGGCGAAC-3', XhoI site is underlined) (Oligomer, Finland). The EcoRI- and XhoI-digested PCR fragment was ligated into the pET28b+ expression vector (Novagen) and the resulting recombinant plasmid (pKTH5316) then propagated in the *E. coli *strain BL21 (DE3)/pLysS for cytosolic production of C-terminal hexahistidine-tagged protein. Recombinant Msp1 (48.4 kDa) also consists of an additional seven N-terminal- and two C-terminal-located amino acids, all of which originate from the coding sequence of the expression vector. Recombinant Msp1 production was carried out as described previously [17]. Briefly, *E. coli *cells with pKTH5316 were cultivated at 37°C in LB-kanamycin (50 μg/ml) broth, and at the mid-log phase they were then induced by 1 mM isopropyl β-D-1-thiogalactopyranoside (IPTG) and grown for another 3 h to allow the accumulation of recombinant Msp1. After cells were pelleted by centrifugation, they were sonicated in lysis buffer (50 mM NaH_2_PO_4 _[pH 8.0], 300 mM NaCl, 10 mM imidazole) and the broken cell suspension that resulted was then clarified by centrifugation and membrane-filtering (0.45-μm pore-size). The clarified suspension was added to a nickel nitrilotriacetic acid (Ni-NTA) agarose (Qiagen) column and, following rinsing with wash buffer (50 mM NaH_2_PO_4 _[pH 8.0], 300 mM NaCl, 20 mM imidazole), recombinant Msp1 was recovered with elution buffer (50 mM NaH_2_PO_4 _[pH 8.0], 300 mM NaCl, 250 mM imidazole). Elution buffer was exchanged to 10 mM Tris-HCl (pH 8.0) with a Bio-Rad EconoPac 10 DG desalting column and purified Msp1 was then concentrated using a 30-kDa Microsep filter (Pall Life Sciences).

### Production of anti-Msp1 serum

Polyclonal antiserum was raised against purified recombinant Msp1 in rabbit as described previously [17]. In brief, a 1:1 mixture of 400 μg Msp1 and Freund's complete adjuvant (1-ml volume) was first administered subcutaneously and then followed by additional 1 ml booster injections (200 μg Msp1 and Freund's incomplete adjuvant as a1:1 mix) every 3 weeks for a total of 9 weeks. Fourteen days after the final booster injection, blood was recovered for antiserum preparation as described elsewhere [26].

### Western blot

For Western blot experiments, equivalent amounts, determined by a bicinchoninic acid (BCA) assay, of protein were separated by SDS-PAGE and subsequently electroblotted onto polyvinyldifluoride (PVDF) membrane. The PVDF sheets were blocked with 3% bovine serum albumine (BSA) in TBS (20 mM Tris-HCl, 500 mM NaCl, pH 7.5) and incubated at room temperature with Msp1 antiserum (1:7500 to 1:15000) for 1 h. After several washings with TBS, blots were incubated for 1 h with goat anti-rabbit antibodies conjugated with alkaline phosphatase (Sigma) at a dilution of 1:1000. Detection was performed with nitro blue tetrazolium and bromo-chloro indolyl phosphate as substrate and the blue coloring reaction was monitored.

### Isolation of different protein fractions from LGG

**(1) supernatant**. LGG bacteria were grown for 24 h in AOAC-medium. After centrifugation (6000 g, 20 min), proteins were precipitated from supernatant by incubation at 4°C for 30 min in the presence of trichloro acetic acid (20% final concentration). After centrifugation (9000 g, 20 min), precipitated proteins were washed twice with cold acetone. Pellet was air-dried and proteins were resuspended in lysis buffer (2 M thio-urea, 7 M urea, 4% CHAPS, 2% DTT). **(2) cell wall**. After centrifugation (6000 g, 20 min) and washing with PBS, bacteria were sonicated in PBS buffer (Branson sonifier) until lysis was observed (several minutes at 10% power). Lysate was cleared by centrifugation (3 times at 4000 g). Cell wall proteins were precipitated by ultracentrifugation (22000 g for 45 min). Pellet was washed 3 times with 50 mM Tris pH8, 500 mM NaCl and dissolved in a small volume PBS. (3) **cytosol**. After centrifugation (6000 g, 20 min), the pellet was washed 2× with 0.9% NaCl. Subsequently, the pellet was dissolved in 1 ml 100 mM Tris, 1% SDS, pH 9.5 and sonicated for 3 min (0.5" on, 1.5" off) with a Branson sonifier. Subsequently, cell debris was removed by centrifugation and proteins were precipitated with TCA precipitation, two times washed with ice-cold 100% acetone, air-dried and then dissolved in PBS.

### Purification of the *L. rhamnosus *GG Msp1 protein

The p75/Msp1 protein was partially purified by cationic exchange as described previously [16]. Hereto, *L. rhamnosus *GG was grown for 24 h in AOAC medium, after which spent culture medium was collected (6000 g, 20 min). The culture supernatant was then loaded onto a SP Sepharose HighPerfomance column (GE Healthcare), equilibrated with 60 mM lactate buffer pH 4.0. Bound proteins were eluted using lactate buffer containing sequential NaCl concentrations (100-1000 mM). Fractions positive for the presence of Msp1 were identified using SDS-PAGE and spin concentrated using Vivaspin filters with MW cut off 10.000 (Sartorius Stedim biotech GmbH, Germany). For the MS/MS experiments, Hi-trap ConA 4B prepacked columns (GE Healthcare) were used according to manufacturer's instructions to further purify positive fractions.

### Characterization of glycan nature of the p75/Msp1 protein

**(i) ProQ Emerald glycoprotein stain**. Samples were loaded onto the wells of the SDS-containing gel after adding buffer and reducing agent according to manufacturer's instructions from the Pro-Q Emerald 488 Glycopotein Gel and Blot stain kit (Molecular Probes, Invitrogen). **(ii) Treatment with TFMS**. Msp1 was chemically deglycosylated by the TFMS method as described before [27] at -20°C for 30 min. After treatment, the proteins were extensively dialyzed and then analyzed by SDS-PAGE. **(iii) Lectin blotting**. The proteins were electroblotted onto PVDF membrane similar as for Western blot. After blocking, the PVDF sheets were incubated at room temperature with lectins conjugated with biotin (biotinylated ConA, WGA or *Ulex europaeus*, Sigma-Aldrich or GNA, HHA, MAA

SNA-I purified and biotinylated according to [20] for 1 h at final concentrations of 0.25 μg/ml in the presence of 1 mM Mg^2+ ^and 1 mM Ca^2+ ^ions. After washing, blots were incubated for 1 h with streptavidin conjugated with alkaline phosphatase (Roche) at a dilution of 1:1000. Detection was performed as described for Western blotting.

### MS determinations

The LGG Msp1 protein purified by cation exchange chromatography was applied to SDS-PAGE using Nu-Page 4-12% gradient Bis-Tris (Invitrogen) gels. The excised gel pieces containing Msp1 were reduced, alkylated and digested with trypsin (Promega), as previously described [28] or with Proteinase K (from *T. album*; Sigma-Aldrich) [21]. After digestion, (glyco-)peptides were collected using two rounds of extraction with 20 μl of 0.1% trifluoroacetic acid and stored at -20°C prior to analysis by mass spectrometry. The total tryptic digest of Msp1 was applied to a reverse phase column (PepMap, 3 μm, 75 μm·x 100 mm; Dionex/Thermo Fisher, Waltham, MA) using an Ultimate 3000 nano-LC system (Dionex/Thermo Fisher). The column was equilibrated at RT with eluent A (0.1% formic acid in water) at a flow rate of 300 nl/min. After injection of the sample a linear gradient was applied (15 min 25% eluent B (95% acetonitrile), 25 min 70% B, 30 min 70% B). The eluate was monitored by absorption at 215 nm. The LC column was coupled to an Esquire HCT-Ultra ESI-ion trap-MS (Bruker Daltonics, Bremen, Germany) equipped with an online nanospray source operated in the positive ion mode. For electrospray (1100-1250 V), electropolished, stainless steel LC-MS emitters (150 μm OD, 30 μm ID) from Proxeon A/S (Odense, Denmark) were used. The solvent was evaporated at 170°C employing a nitrogen stream of 6 l/min. When operated in the auto MS/MS mode, monitoring ions from *m*/*z *500 to 1600, each MS scan was followed by the acquisition of MS/MS spectra of up to five of the most abundant ions in the MS spectrum.

The digest generated by Proteinase K was separated using an Ultimate 3000 nano-LC system (Dionex/Thermo Fisher) equipped with a graphitized carbon trap column (Hypercarb, 5 μm, 170 μm × 10 mm; Thermo Scientific) and nano column (75 μm x·100 mm) packed by Grom Analytik (Rottenburg, Germany). The column was equilibrated at RT with eluent A (0.1% formic acid in water) at a flow rate of 400 nl/min. After injection of the sample a linear gradient was applied (15 min, 25% eluent B (95% acetonitrile); 25 min, 70% B; 30 min, 70% B). The eluate was monitored by absorption at 215 nm. Eluates were analyzed by ESI-ion trap-MS as described above. The ions were monitored from *m*/*z *300 to *m*/*z *1500 in the MS mode. The MS/MS spectra were analyzed with Data Analysis (Bruker Daltonics), converted to MGF files and searched against NCBI database, with search limitation "bacteria" using the MASCOT search algorithm. Carbamidomethylation of cysteines was set as a fixed modification and oxidized methionines were set as a variable modification. Trypsin was specified as the proteolytic enzyme and missed cleavages were not allowed. The mass tolerance of the precursor ion was set to 2 Da and that of the fragment ions was set to 0.5 Da. In addition, MS/MS spectra were used for manual interpretation and *de novo *sequencing.

### Activation of Akt in Caco-2 cells

Caco-2 cells were cultured as described before [15]. 24 h before the experiments, the Caco-2 cells were deprived of FBS. LGG wild-type was grown overnight in AOAC medium, centrifuged at 4000 × g and 4°C for 10 min, and washed with cold PBS. Caco-2 cells were incubated with 1.5 ml of DMEM without FBS containing either 1 × 10^7 ^CFU/ml LGG wild-type or *msp1 *mutant CMPG10200, 100 ng/ml wild-type, recombinant or TFMS-treated Msp1. DMEM without FBS was used as a negative control. After 16 h incubation at 37°C, cell lysates were made with HEPES-lysis buffer containing 25 mM HEPES pH 7.5, 0.3 M NaCl, 1.5 mM MgCl_2_, 20 mM β-glycerol-phosphate, 2 mM EDTA, 2 mM EGTA, 1 mM DTT, 1% (v/v) Triton X-100, 10% (v/v) glycerol, 10 μg/μl leupeptin, 5 μg/μl aprotinin, 1 mM PMSF, 1 mM Na_3_VO_4 _and 50 mM NaF. Protein concentration was determined using BCA assay (Perbio, Thermo Fisher Scientific). Samples with loading buffer were prepared and processed on the CriterionTM system (Bio-Rad Laboratories, Hercules, CA, USA) on a 4-12% Bis-TRIS gel (Biorad) and Protran 2 μm-pored nitrocellulose membrane (Perkin-Elmer, Wellesley, MA, USA). Membranes were blocked for 1 h at room temperature in Tris-buffered saline containing 0.1% Tween-20 and 5% BSA. The membrane was incubated overnight at 4°C with the primary antibody diluted in 5% BSA in 1× TBS plus 0.1% Tween 20, then washed 3 times with TBS plus 0.1% Tween 20, followed by incubation with the second polyclonal antibody for 2 h at 4°C. Antibodies against phospho-AKT (Ser473) and total AKT were obtained from Cell Signaling Technology (Beverly, MA).

### Zymogram analysis

The cell wall hydrolyzing activity was investigated by zymogram analysis as described previously by Lepeuple *et al. *(14). SDS-PAGE was performed with 10% (w/v) polyacrylamide separating gels (NuPage, Invitrogen). Autoclaved LGG cells treated with 10% TCA were added to the gels as enzyme substrates and the gels were loaded with 10 to 20 μg of protein samples. After sample migration, the gels were washed for 30 min in deionized H_2_O at room temperature and then incubated in a phosphate buffer, pH 6.2, 1 mM dithiothreitol (DTT) containing 0.1% (v/v) Triton X-100, overnight at 37°C. The gels were subsequently washed for 30 min in deionized H_2_O, then stained with 0.1% methylene blue in 0.01% (w/v) KOH for 2 h at room temperature and destained in deionized H_2_O.

## List of abbreviations

DMEM: Dulbecco's Modified Eagle Medium; ConA: concanavalin A; CW-PS: cell wall polysaccharides; EPS: exopolysaccharides; ESI: electrospray ionization; GNA: *Galanthus nivalis *lectin; HHA: *Hippeastrum hybrid *lectin; IEC: intestinal epithelial cell; LC: liquid chromatography; LGG: *Lactobacillus rhamnosus *GG; MAA: *Maackia amurensis *lectin; MS: mass spectrometry; Msp: major secreted protein; RP: reverse phase; SN: supernatant; SNA-I: *Sambucus nigra *lectin I; TFMS: trifluoromethanesulfonic acid; WGA: wheat germ agglutinin

## Competing interests

The authors declare that they have no competing interests.

## Authors' contributions

SL designed and performed part of the experiments, analyzed the data and wrote the paper. IJJC constructed the *msp1 *mutant, carried out part of the zymogram analysis and participated in the analysis of the data and editing of the paper. CIAB performed the MS analyses and participated in the analysis of the data and editing of the paper. GS was responsible for the proteomic experiments in this study. TLAV carried out part of the molecular DNA work. HLPT participated in the glycoproteomic analyses. IvO was responsible for the expression of recombinant Msp1 protein and production of anti-Msp1 serum and also participated in the writing of the paper. KN and PA participated in the Akt signaling analysis. EJMVD provided some lectins used in this study and useful comments for glycoprotein detection. WMdV, AP and AMD participated in the design of the study. MW, SCJDK and JV participated in the design and coordination of the experiments, analysis of the data and writing of the manuscript. All authors read and approved the final manuscript.
